# Development and validation of the Japanese version of EPDS‐P for indirect screening of paternal perinatal depression based on maternal reporting: Protocol for a prospective longitudinal observational study

**DOI:** 10.1002/npr2.12321

**Published:** 2023-01-25

**Authors:** Keita Tokumitsu, Norio Yasui‐Furukori, Sheehan David Fisher, Takako Keta, Chihiro Yamada, Junko Takeuchi, Koji Yachimori, Norio Sugawara, Kazutaka Shimoda

**Affiliations:** ^1^ Department of Psychiatry Dokkyo Medical University School of Medicine Tochigi Japan; ^2^ Department of Neuropsychiatry Towada City Hospital Towada Japan; ^3^ Department of Psychiatry and Behavioral Sciences Northwestern University, Feinberg School of Medicine Chicago Illinois USA; ^4^ Parent and Child Support Section Towada City Community Health Center Towada Japan

**Keywords:** EPDS‐P, men, partner, perinatal depression, screening

## Abstract

**Aims:**

The main purpose of this study is to develop an indirect screening system for paternal perinatal depression based on the female partner's assessment in the Japanese population. The Japanese version of the Edinburgh Postnatal Depression Scale‐Partner (EPDS‐P) will be used as the indirect screening tool, and its accuracy will be studied in this longitudinal prospective observational study.

**Methods:**

Public health nurses and midwives at the participating community health center are currently inviting couples to participate, and are distributing self‐rating scales to the participants. The primary evaluation scales being used in this study are the Japanese versions of the Center for Epidemiologic Studies Depression Scale (CES‐D) and the Japanese version of the EPDS‐P which evaluates paternal perinatal depression by women. We will evaluate EPDS‐P performance against CES‐D, including accuracy, sensitivity, specificity, and correlations.

**Results and Conclusions:**

Perinatal depression is a mental illness that occurs between pregnancy and postpartum within the 12 months, and it is known to increase the risk of adversely impacting on child development. Men may also experience a psychosocial crisis during their partners' perinatal period. Although it was recently reported that the EPDS‐P can indirectly detect paternal perinatal depression, there is, as yet, insufficient evidence of this because the previous studies had relatively small sample sizes and were limited to cross‐sectional studies in the postpartum period. The development of a screening system for paternal perinatal depression using the EPDS‐P will lead to increased awareness of the disease and provide an opportunity to establish a family‐based support system in Japan.

## Abstract 2

The main purpose of this study is to develop an indirect screening system for paternal perinatal depression based on the female partner's assessment in the Japanese population. The Japanese version of the Edinburgh Postnatal Depression Scale‐Partner (EPDS‐P) will be used as the indirect screening tool, and its accuracy will be studied in this longitudinal prospective observational study.

## Graphical abstract

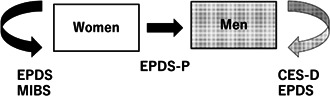

## 1 INTRODUCTION

Perinatal depression is a psychiatric disorder that occurs during pregnancy and within the first year postpartum, and it is known to increase the risk of adverse impacts on child development.^1^, ^2^, ^3^, ^4^ Known causes of maternal perinatal depression include biological, psychological, and social problems, with family support having a strong positive impact on women's mental health.^5^ For this reason, men are generally encouraged to assist women and children during the perinatal period.^6^ It has become clear that men may also experience psychosocial crises and high rates of depression during the perinatal period.^7^ An international meta‐analysis found the prevalence of paternal perinatal depression to be 8.4%.^8^ In addition, paternal perinatal depression is known to increase the risk of suicide in men.^9^ Our previous meta‐analysis found the prevalence of perinatal depression among Japanese men to be approximately 10%, with no statistically significant difference in prevalence between men and women in terms of relative risk.^10^ Risks of maternal and paternal perinatal depression as well as child abuse are reported to correlate with each other.^9^, ^11^ This suggests that supporting the father might ultimately have a positive impact on the mental stability of the mother and on the development of the child.^12^ Therefore, it is necessary to assess the mental state of men as well as of women, and to provide appropriate support during the perinatal period. However, men are less likely to participate in the perinatal support system than are women.

In addition, health care professionals have tended to overlook the fact that men can also experience perinatal depression, and Japan lacks systems for screening and prevention of paternal perinatal depression.^13^ These factors make it difficult to detect paternal perinatal depression in Japanese men.^14^ Furthermore, inadequate knowledge of paternal postpartum depression among health care professionals might be a barrier to appropriate treatment.^15^ For this reason, more attention should be paid to paternal perinatal depression during routine perinatal checkups. In addition, providing information and support for men about paternal perinatal depression using an online screening and support system is also important because it offers the advantage of easy access to information with fewer human and physical constraints^16^, ^17^ but not yet widespread enough in Japan. Therefore, new systems to support men's mental health during the perinatal period are being considered. Unfortunately, a previous Japanese study suggested that the validity of the Japanese versions of the Kessler Psychological Distress scales, K6^18^ and K10,^19^ and the Patient Health Questionnaire‐9 (PHQ‐9)^20^ might be compromised when used by women to assess perinatal depression in their male partners.^21^

For the assessment of perinatal depression, the Edinburgh Postnatal Depression Scale (EPDS), a 10‐item self‐rating scale devised by Cox et al.^22^ is commonly used. The Japanese version of the EPDS has also been validated and widely used.^23^ In recent years, the EDPS‐Partner (EPDS‐P) has been developed to assess perinatal depression based on the EPDS, and studies have indirectly assessed perinatal depression in men through women.^24^ The EPDS‐P can indirectly screen fathers for perinatal depression through maternal reports, even when fathers do not participate in perinatal maternal and child support services or when a coronavirus pandemic makes it difficult for fathers and mothers to access healthcare resources. The EPDS‐P can indirectly assess postpartum depression in men with a sensitivity of 89% and specificity of 59% with a cutoff value of 4/5 in the general population of the United States; thus, it is attracting attention as a useful assessment tool.^24^ However, the EPDS cut‐off value varies across countries and cultures, and studies have yet to examine the validity of the EPDS‐P for perinatal depression in Japanese men. Also, few studies have examined the longitudinal availability of the EPDS‐P from pregnancy to the postpartum period.^24^ We therefore planned this prospective observational study to examine the validity of the Japanese version of the EPDS‐P to indirectly screen paternal perinatal depression in Japanese men for application to real‐world clinical practice. We believe that the development of a screening system for paternal perinatal depression using the EPDS‐P will lead to increased awareness of the disease and provide an opportunity to establish a family‐based support system in Japan.

### 1.1 Objective

The main purpose of this study is to develop an indirect screening system for paternal perinatal depression based on the female partner's assessment in the Japanese population. The Japanese version of the EPDS‐P will be used as the indirect screening tool, and its accuracy will be studied in this longitudinal prospective observational study. Additionally, we will also investigate whether there is a correlation between depressive symptoms and risk of child maltreatment in women and men in our study participants.

## 2 METHODS AND ANALYSIS

### 2.1 Study design and subjects

This study is a prospective longitudinal observational study of the participating couples in Towada City, Japan. Research participants are being recruited during perinatal home visits by public health nurses and midwives from a community health center in Towada City, Aomori Prefecture, Japan. All research participants will receive a full explanation of the study and provide written informed consent. The mental status of the participants will be assessed using self‐rating scales. Assessments will be conducted at two points: late in pregnancy and within 3 months postpartum. We will provide potential study participants with information about perinatal depression, regardless of their rating scale scores or consent to participate in the study. We will also explain that support from public health nurses and treatment from physicians will be available. For residents at high risk of perinatal depression such as high EPDS and CES‐D scores, poor childcare support, and extreme economic deprivation, we will develop support through collaboration between health centers and medical institutions.

The target population is the general public of Towada, a city located in the southern part of Aomori Prefecture, Japan, with a population of approximately 60 000. Public health nurses or midwives at the Towada Community Health Center are carrying out the recruitment of research participants, and they have sufficient experience in perinatal maternal and child support, and have received research ethics training beforehand. The explanatory document and questionnaire have been prepared in Japanese, and participants who cannot understand Japanese will be excluded from the study. Participants under 20 years of age will also be excluded from the study because they cannot legally consent to the study.

### 2.2 Data collection

#### 2.2.1 Instruments

The following screening instruments are being used for data collection:

- The Center for Epidemiologic Studies Depression Scale (CES‐D)
  This scale was developed by the National Institute of Mental Health (NIMH) for epidemiological studies to detect depression in the general population.^25^ The CES‐D is a 20‐item self‐rating questionnaire that assesses symptoms of depression. The cut‐off value is 16 points, and a Japanese version of the CES‐D (CES‐D‐J) has been validated.^26^
- The Edinburgh Postnatal Depression Scale (EPDS)
  The EPDS is a 10‐item self‐rating scale designed by British psychiatrist John Cox to screen for postpartum depression.^22^ The Japanese version of the EPDS was validated in 1996.^23^ Recently, the EPDS has also been shown to be a reliable and valid measure of maternal prenatal depression^27^ and is attracting attention as a rating scale for paternal perinatal depression.^8^ The diagnostic accuracy of the EPDS for postpartum depression in Japanese men has been reported to have a sensitivity of 81.8% and a specificity of 94.1% in the postpartum period, with a cutoff value of 8,^28^ but a similar study is lacking for the prenatal period.
- The Edinburgh Postnatal Depression Scale‐Partner (EPDS‐P)
  In regard to the original English‐language versions, the EPDS‐P was developed to assess postpartum depression in women from man’ perspective.^29^ In contrast, the EPDS‐P developed by Fisher et al.^24^ assesses paternal postpartum depression through maternal reporting. The diagnostic accuracy of the EPDS‐P has been reported to have a sensitivity of 89% and a specificity of 59% with a cutoff value of 4/5, but a Japanese version had yet to be developed. Therefore, with the help of Dr. Fisher, the English version of EPDS‐P for paternal depression was translated into Japanese, and back‐translated into English by three native speakers of Japanese and English and validated its Japanese version.
- The Mother‐to‐Infant Bonding Scale (MIBS)
  The MIBS is a self‐rating questionnaire that assesses a mother's feelings toward her infant.^30^ The MIBS includes 10 items that assess the mother–child bond.^31^ Each item is rated on a four‐point Likert‐type scale (0–3). The higher the MIBS score, the weaker the mother–child bond. The Japanese version of the MIBS has been previously validated and is commonly used in Japan to assess mother–child bonding.^31^

**FIGURE 1:**
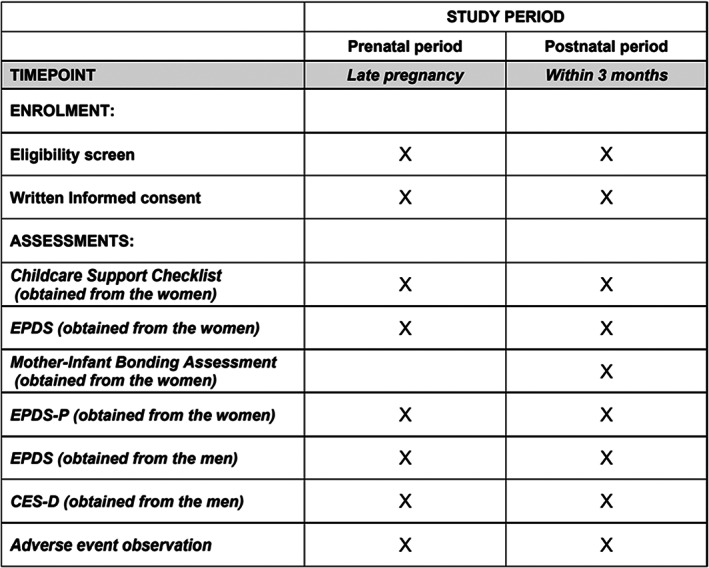
SPIRIT schedule. CES‐D, Center for Epidemiologic Studies Depression Scale; EPDS, Edinburgh Postnatal Depression Scale; EPDS‐P, Edinburgh Postnatal Depression Scale‐Partner.

**FIGURE 2:**
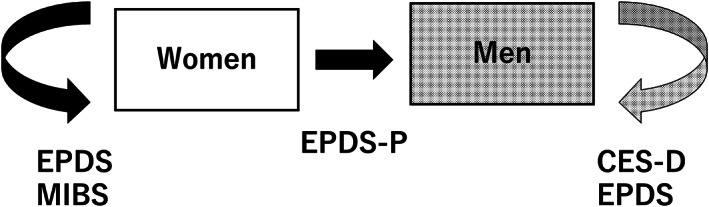
Evaluation Direction of Rating Scales. CES‐D, Center for Epidemiologic Studies Depression Scale; EPDS, Edinburgh Postnatal Depression Scale; EPDS‐P, Edinburgh Postnatal Depression Scale‐Partner.

Health workers and midwives do not need to be trained to explain to participants how to use the screening instruments EPDS, EPDS‐P, and MIBS. Public health nurses and midwives from Towada Community Health Center are visiting the homes of expectant and perinatal mothers to evaluate the mental status of study participants using these rating scales. They are informing study participants in advance of the study's purpose and the voluntary and anonymous nature of their participation, and are obtaining written informed consent from all participants in the study. Female study participants will complete the EPDS, EPDS‐P, and MIBS. Male study participants will also complete the CES‐D and EPDS. Also, participants will be asked to answer questionnaire items about their age, gender, parity, childcare concerns, loss of a child within 1 year or not, satisfaction with their living environment, and social support status (Figures 1 and 2).

### 2.3 Outcome measures

The primary outcome will be accuracy of the EPDS‐P in determining paternal perinatal depression in Japanese men. Presence of paternal depression is determined based on the CES‐D, with a CES‐D total score of 16 or higher considered depression.

Then, ROC curves are drawn and AUCs are calculated to examine the discrimination accuracy of the two groups (depression or not) by EPDS‐P. Discriminatory value of ROC curves was interpreted as excellent (AUC 0.9–1), good (0.8–0.89), fair (0.7–0.79), poor (0.6–0.69), or fail/no discriminatory capacity (0.5–0.59).^32^ On the other hand, the point on the ROC curve where “sensitivity + specificity” is maximized is defined as the optimal cut‐off point. The sensitivity and specificity at the cutoff point are calculated, respectively.

The secondary outcome will be whether prenatal EPDS‐P can predict paternal postpartum depression using longitudinal data from pregnancy and the early postpartum period. Furthermore, we will calculate the correlation between the EPDS and CES‐D in men and analyze the performance of the EPDS in identifying paternal perinatal depression.

### 2.4 Sample size calculation

A meta‐analysis of reports based on self‐rating scales estimated the prevalence of paternal perinatal depression among Japanese men to be approximately 10%.^10^ Therefore, the sample size required to validate the accuracy of the EPDS‐P in the last trimester of pregnancy and within 3 months postpartum was estimated to be 554 at each time point, assuming a standard normal distribution, 95% confidence level, 0.025 margin of error (α/2), and 10% estimated value of the population proportion. Assuming a valid response rate of 90%, the target number of cases was set at 600 pairs for each time‐point. In Towada City, public health nurses have been making home visits to nearly all expectant and nursing mothers as part of the routine work of the maternal and child health care system since before this study began. An interview survey including the EPDS was conducted with women visited by these public health nurses, and cooperation was obtained in all cases. For this reason, we assumed that a high percentage of women would cooperate in the screening survey for paternal perinatal depression and therefore assumed the rate to be 90%. Given approximately 300 births per year and an extremely high rate (>95%) of home visits by public health nurses and midwives to expectant mothers in Towada City, the target number is expected to be achieved within a 30‐month survey period.

### 2.5 Data analysis plan

Upon completion of data collection, the database will be locked for statistical analysis. First, we will calculate the screening accuracy of the EPDS‐P, which in this study assesses depressive symptoms in men by women.

Men with a CES‐D score of 16 or higher are defined as the “depressed” group, and those with a CES‐D score of 15 or lower are defined as the “non‐depressed” group. ROC curves are drawn and AUCs are calculated to examine the discrimination accuracy of the two groups by EPDS‐P score. The discriminatory value of the ROC curves is interpreted as follows: excellent (AUC 0.90–1.00), good (0.80–0.89), fair (0.70–0.79), poor (0.60–0.69), or fail/no discriminatory capacity (0.5–0.59).^32^ The point on the ROC curve where sensitivity + specificity is maximized is defined as the optimal cut‐off point, and the sensitivity and specificity at the cut‐off point are calculated separately.

Next, we will determine the absolute value of Spearman's non‐parametric correlation coefficient between the EPDS‐P and CES‐D. We will also set men scoring 16 or higher on the CES‐D as the depressed group,^26^ and then perform a receiver operating characteristic (ROC) curves analysis to calculate optimal cut‐off values and sensitivity and specificity of the EPDS‐P. We also plan to examine the reliability of the EPDS‐P by calculating its Cronbach's alpha. Cronbach's alpha is the most widely used objective measure of reliability. Alpha is an important concept in the evaluation of assessments and questionnaires. Cronbach's alpha is a confidence coefficient that evaluates whether or not the items measure the same concept or object as a whole (internal consistency) when multiple questions are asked about a characteristic, and the sum of the responses is used as the characteristic scale.^33^ If the Cronbach's alpha coefficient for each item is above 0.6, it is considered to have good internal stability, reliability, and acceptable consistency for an exploratory study.^34^

The cut‐off value of the EPDS‐P in detecting paternal depression will be calculated at two points: in late pregnancy and within 3 months postpartum. The statistical analysis did not include any participant's response having missing values or an inappropriate number of answers to a question item on a rating scale. Then, as a secondary analysis, we would like to stratify depressed and non‐depressed women, analyze the accuracy of the EPDS‐P in identifying paternal perinatal depression in each group, and examine the impact of women's mental status on the accuracy of the EPDS‐P.

In addition, we will examine the absolute value of Spearman's non‐parametric correlation coefficient between the CES‐D and EPDS in men and the accuracy of the EPDS in identifying depression in men. The depression group is defined as those with a CES‐D score of 16 or higher. Based on this, participants are divided into a depression group or a non‐depression group. We will also report the sensitivity and specificity of the cut‐off points for the EPDS in men at the pregnancy and postpartum. The global performance of the EPDS in men will be expressed as area under the ROC curve.

We will also analyze the characteristics of participants who first developed depression after childbirth.

As with maternal depression, paternal postpartum depression might develop during pregnancy, so we will calculate the proportion of cases of paternal postpartum depression that develop throughout the prenatal to postpartum period.

We will examine whether EPDS‐P scores during pregnancy predict paternal postpartum depression. First, postpartum men will be divided into two groups: a depressed group (CES‐D total score of 16 or higher) and a non‐depressed group (CES‐D total score of 15 or lower). We will then draw an ROC curve using the EPDS‐P score during pregnancy and calculate the area under the ROC curve (AUC). The discriminatory value of the ROC curves will be interpreted as follows: excellent (AUC 0.90–1.00), good (0.80–0.89), fair (0.70–0.79), poor (0.60–0.69), or fail/no discriminatory capacity (0.50–0.59).^32^ If the AUC value is large, it is considered that the EPDS‐P score during pregnancy can discriminate paternal postpartum depression because EPDS‐P score during pregnancy can predict paternal postpartum depression. We will then determine the EPDS‐P optimal cutoff value during pregnancy that most accurately identifies paternal postpartum depression (the point on the ROC curve where sensitivity + specificity is maximized).

Furthermore, we will use a covariance structure analysis and binomial logistic regression analysis to test whether maternal EPDS score, MIBS score, and other maternal characteristics affect their EPDS‐P scores. All statistical analyses will be performed using IBM SPSS 28 and Amos 28 (IBM Japan, Tokyo, Japan).

### 2.6 Patient and public involvement

Research participants and the general public were not involved in the development of the research questions or in the design of this study. All participants and interested parties will receive a summary of the results from the principal investigator upon request.

Contact information for our ethics committee: Institutional Review Board of the Ethics Committee of the Dokkyo Medical University; 880 Kitakobayashi, Shimotsugagun, Mibu, Tochigi, Japan, Postal Code 321‐0293, Phone: +81‐282‐86‐1111.

### 2.7 Duration and current status of the study

This study protocol was registered with the University Hospital Medical Information Network‐Clinical Trials Registry: UMIN‐CTR (https://www.umin.ac.jp/ctr/) on September 28, 2021 (Trial registration number: UMIN000045584). UMIN‐CTR has been recognized by the International Committee of Medical Journal Editors (ICMJE) as an “acceptable registry.”

The survey was initiated on October 1, 2021 in Towada City, Aomori Prefecture, Japan. The study is being conducted at two timepoints: in late pregnancy and within 3 months postpartum. Recruitment of participants is ongoing. As of December 31, 2021, no interim analyses have been conducted. The study is scheduled to end on March 31, 2024.

### 2.8 Ethics and dissemination

#### 2.8.1 Ethical aspects and informed consent

This research protocol was peer‐reviewed and approved by the Ethical Review Board of Dokkyo Medical University on September 3, 2021 (Approval No. 2021‐015). This research will be conducted in accordance with the Declaration of Helsinki and the Ethical Guidelines for Medical and Biological Research Involving Human Subjects as certified by the Japanese Ministry of Education, Culture, Sports, Science and Technology (MEXT), the Ministry of Health, Labour and Welfare, and the Ministry of Economy, Trade and Industry. Any protocol amendments (changes to eligibility criteria, outcomes, analyses) that are necessary will be communicated and modified in the relevant parties.

Research participants will be informed of the research in advance, both in writing and verbally. In addition, all participants will participate in the study after providing written informed consent. We emphasize to the research participants that their participation in the study is completely voluntary and that they can withdraw their consent at any time. In this study, public health nurses and midwives from the Towada Community Health Center are conducting sampling during perinatal home visits. All staff involved in the study have attended a research ethics course. Different researchers are in charge of the sampling and data analysis, and efforts are being made to eliminate analytical bias. In addition, this study does not prevent participants who notice their own need for treatment of perinatal depression from visiting the Department of Psychiatry at Towada City Hospital or Dokkyo Medical University, the institutions where the study is being conducted.

Anonymized information will be used in the preparation and analysis of data sheets. Participants will have their personal data protected and their identification code entered as the participant identification number for the research. The data collected in this study were entered into an Excel spreadsheet and saved with password protection. Information that could identify individuals, such as name and address, were not entered into the spreadsheet. The participant's identification code was entered as the participant identification number for the research. The Excel data will be stored on computers not connected to the Internet at the Department of Psychiatry at Dokkyo Medical University Hospital as well as Towada City Hospital, and the Parent and Child Support Section of the Towada City Community Health Center. After the completion of the study, the data will be stored for 10 years and then promptly deleted and destroyed.

A list of participants will be prepared on paper, with only their first and last initials and participant identification codes written. This list will not be converted to electronic media and will be strictly managed by the Department of Neuropsychiatry, Towada Hospital, and the Parent and Child Support Section of the Towada City Community Health Center.

#### 2.8.2 Dissemination plan

Study results will be disseminated through publication in international peer‐reviewed journals and conference presentations to psychiatric and perinatal health professionals. The early detection and treatment of paternal perinatal depression using the Japanese version of the EPDS‐P is expected to lead to perinatal support not only for men but also for women and children from the perspective of a family‐based support system. The establishment of a new screening system for paternal perinatal depression is also expected to have a substantial impact on the welfare policy and medical education in Japan. Confirming the usefulness of the EPDS‐P for indirect screening of Japanese men for paternal perinatal depression is expected to lead to more efficient use of limited health and welfare resources.

### 2.9 Strengths and limitations

This longitudinal observational study has a sufficient sample size compared with previous reports. A self‐rating scale is being used to assess the study participants, and the screening system using the self‐rating scales does not require technical proficiency to administer and answer, which makes it easy to apply in real‐world clinical practice. However, as a regional study, our findings might not be generalizable to other regions. In addition, assessment of perinatal depression using a self‐rating scale differs from psychiatric diagnosis based on a structured interview, and may not accurately reflect the mental status of the participants, and might include false positives and false negatives of depression.

## AUTHOR CONTRIBUTIONS

Keita Tokumitsu: study conception and design, writing the first draft manuscript. Takako Keta and Chihiro Yamada: data collection. Norio Yasui‐Furukori, Sheehan David Fisher, Junko Takeuchi, Koji Yachimori, Norio Sugawara, and Kazutaka Shimoda: critical review of the manuscript.

## FUNDING INFORMATION

The Japan Society for the Promotion of Science (JSPS), under the jurisdiction of the Ministry of Education, Culture, Sports, Science and Technology (MEXT), reviewed this research plan and provided a Grant‐in‐Aid for Scientific Research (KAKENHI, number 21K10503 to Principal Investigator Keita Tokumitsu). The funder had no role in the study design, the data collection and analysis, the decision to publish, or the preparation of the manuscript.

## CONFLICT OF INTEREST

Norio Yasui‐Furukori has been a speaker for Dainippon‐Sumitomo Pharmaceutical, Mochida Pharmaceutical, and MSD. Kazutaka Shimoda has received research support from Meiji Seika Pharma Co., Pfizer Inc., Dainippon Sumitomo Pharma Co., Ltd., Daiichi Sankyo Co., Otsuka Pharmaceutical Co., Ltd., Astellas Pharma Inc., Novartis Pharma K.K., Eisai Co., Ltd., Takeda Pharmaceutical Co., Ltd. and honoraria from Mitsubishi Tanabe Pharma Corporation, Meiji Seika Pharma Co., Ltd., Dainippon Sumitomo Pharma Co., Ltd., Takeda Pharmaceutical Co., Shionogi & Co., Ltd., Daiichi Sankyo Co., Pfizer Inc. and Eisai Co., Ltd. These companies had no role in the study design, the data collection and analysis, the decision to publish, or the preparation of the manuscript. The remaining authors declare that they have no competing interests to report.

## ETHICAL APPROVAL

This study was conducted in accordance with the Declaration of Helsinki and the Japanese Ethical Guidelines for Medical and Health Science Research Involving Human Subjects. Prior to the start of this study, the research protocol was reviewed and approved by the Ethical Review Board of Dokkyo Medical University on September 3, 2021 (Approval No. 2021‐015).

## Informed Consent

This study is a prospective longitudinal observational study of the participating couples in Towada City, Japan. Research participants are being recruited during perinatal home visits by public health nurses and midwives from a community health center in Towada City, Aomori Prefecture, Japan. All research participants will receive a full explanation of the study and provide written informed consent.

## Registry and the Registration No. of the Study/Trial

N/A.

## Animal Studies

N/A.

## Data Availability

As our research dataset contains potentially sensitive information, we have ethical restrictions. Please contact the Institutional Review Board of the Ethics Committee when requesting data. Contact information for our ethics committee: Institutional Review Board of the Ethics Committee of the Dokkyo Medical University; 880 Kitakobayashi, Shimotsugagun, Mibu, Tochigi, Japan, Postal Code 321‐0293, Phone: +81‐282‐86‐1111.
